# 气相色谱-四极杆/飞行时间质谱筛查确证辣椒中244种农药残留及其代谢物

**DOI:** 10.3724/SP.J.1123.2020.11019

**Published:** 2021-05-08

**Authors:** Qi CAO, Yazhen ZHANG, Zhengwei ZHU, Wanqin WU, Feng JIANG, Tingting YU

**Affiliations:** 湖北省食品质量安全监督检验研究院, 湖北省食品质量安全检测工程技术研究中心, 湖北 武汉 430075; Hubei Provincial Institute for Food Supervision and Test, Hubei Provincial Engineering and Technology Research Center for Food Quality and Safety, Wuhan 430075, China; 湖北省食品质量安全监督检验研究院, 湖北省食品质量安全检测工程技术研究中心, 湖北 武汉 430075; Hubei Provincial Institute for Food Supervision and Test, Hubei Provincial Engineering and Technology Research Center for Food Quality and Safety, Wuhan 430075, China; 湖北省食品质量安全监督检验研究院, 湖北省食品质量安全检测工程技术研究中心, 湖北 武汉 430075; Hubei Provincial Institute for Food Supervision and Test, Hubei Provincial Engineering and Technology Research Center for Food Quality and Safety, Wuhan 430075, China; 湖北省食品质量安全监督检验研究院, 湖北省食品质量安全检测工程技术研究中心, 湖北 武汉 430075; Hubei Provincial Institute for Food Supervision and Test, Hubei Provincial Engineering and Technology Research Center for Food Quality and Safety, Wuhan 430075, China; 湖北省食品质量安全监督检验研究院, 湖北省食品质量安全检测工程技术研究中心, 湖北 武汉 430075; Hubei Provincial Institute for Food Supervision and Test, Hubei Provincial Engineering and Technology Research Center for Food Quality and Safety, Wuhan 430075, China; 湖北省食品质量安全监督检验研究院, 湖北省食品质量安全检测工程技术研究中心, 湖北 武汉 430075; Hubei Provincial Institute for Food Supervision and Test, Hubei Provincial Engineering and Technology Research Center for Food Quality and Safety, Wuhan 430075, China

**Keywords:** QuEChERS, 气相色谱-四极杆/飞行时间质谱, 农药残留, 数据库, 辣椒, 筛查确证, QuEChERS, gas chromatography-quadrupole time-of-flight mass spectrometry (GC-Q-TOF/MS), pesticide residues, database, chilli, screening and confirmation

## Abstract

建立了辣椒中244种农药残留的QuEChERS前处理结合气相色谱-四极杆/飞行时间质谱(GC-Q-TOF/MS)快速筛查确证方法。鲜辣椒和干辣椒样品分别采用经-20 ℃冷冻的乙腈和1%(v/v)乙酸化乙腈提取,经盐析分层、分散固相萃取净化和浓缩后加入内标并复溶,HP-5MS UI色谱柱(30 m×0.25 mm×0.25 μm)分离,程序升温不分流进样,GC-Q-TOF/MS全扫描模式采集,内标法定量。比较了分析保护剂(AP)和基质匹配校准法对基质效应的补偿效果,最终选择采用基质匹配校准法来补偿基质效应并进行样品中农药残留的校准定量。设置定性筛查中的保留时间最大偏差为±0.25 min,精确质量偏差阈值为±20×10 ^-6^。对鲜辣椒中244种农药残留和干辣椒中222种农药残留进行了定量方法验证,实验结果表明,采用建立的数据库和分析方法可以对辣椒进行农药残留的高通量筛查和定量分析。在空白辣椒样品中添加不同水平的目标化合物,以信噪比*S/N*≥10对应的添加水平作为定量限(LOQ)。鲜辣椒中最大残留限量(MRL)≤0.05 mg/kg的44种农药在鲜辣椒中LOQ≤0.010 mg/kg,线性范围在0.01~1.00 mg/L,在1倍和2.5倍LOQ添加水平下,回收率在60%~120%的农药种类占比分别为88.64%和100%;鲜辣椒中暂无MRL规定或MRL>0.05 mg/kg的200种农药在鲜辣椒中LOQ≤0.025 mg/kg,线性范围在0.05~1.00 mg/L,在1倍、2倍和10倍LOQ添加水平下,回收率在60%~120%的农药种类占比分别为49.50%、87.00%和89.50%; 244种农药的线性相关系数(*r* ^2^)均大于0.99。222种农药在干辣椒中LOQ≤0.15 mg/kg,线性范围在0.04~1.00 mg/L, *r* ^2^≥0.99的比例为95.46%,在1倍、2倍和10倍LOQ添加水平下,回收率在60%~120%占比分别为72.52%、73.42%和81.53%。应用建立的筛查确证方法对市售的12份鲜辣椒样品和14份干辣椒样品进行农药残留筛查分析,从9份鲜辣椒样品和3份干辣椒样品中筛查出8种农药化合物,经人工鉴定均为阳性,定量结果显示,8种农药化合物均未超过其在GB 2763-2019《食品安全国家标准食品中农药最大残留限量》所规定的MRL。方法快速、简单、高效、可靠,适用于鲜辣椒及干辣椒中多种农药残留的筛查分析。

## Abstract (en)

QuEChERS pretreatment combined with gas chromatography-quadrupole time-of-flight mass spectrometry (GC-Q-TOF/MS) has been investigated for application in screening 244 pesticide residues in chilli. Fresh chilli samples were extracted with acetonitrile, and dried chilli samples were extracted using an acetonitrile/acetic acid (99∶1, v/v) mixture. The two extraction solvents were stored at -20 ℃. After salting out and cleaning by dispersive solid phase extraction (dSPE), heptachlor epoxide B was added as an internal standard, and the resulting residues were dissolved in 1.00 mL acetone. The dissolved sample solution was loaded onto an HP-5MS UI column (30 m×0.25 mm, 0.25 μm) and eluted by GC-Q-TOF/MS with a programmable temperature vaporizer and splitless injection in the full-scan mode. The compensation effects of the analytical protectant (AP) and matrix-matched calibration method on the matrix effect were established. AP could be used in the fresh chilli matrix to compensate for matrix effects, but it was not effective in the dried chilli matrix. The matrix-matched calibration method was effective in both matrices, which was selected for the quantification of pesticide residues in the samples. Because of the existence of the isomers of one compound and the same characteristic ions of different compounds, analyte detection was based on a flexible retention time deviation of ±0.25 min and accurate mass deviation of ±20×10 ^-6^. Screening was performed by the software in the automatic matching mode. Compound identification and quantitation were based on a database and calibration curve established with reference materials. Suspicious samples were subjected to manual analysis. Quantitative analysis of 244 pesticide residues in fresh chilli and 222 pesticide residues in dried chilli was performed. The results showed that the developed database and method can provide a reference for the high-throughput screening and quantitation of fresh and dried chilli. Different levels of pesticides were added to the blank chilli samples, and the addition level corresponding to a signal-to-noise ratio (*S/N*) of 10 was used as the limit of quantification (LOQ). The LOQs of 44 pesticides with a maximum residue limit (MRL) ≤0.05 mg/kg in fresh chilli did not exceed 0.010 mg/kg. The linear ranges of these 44 pesticides were 0.01-1.00 mg/L. At spiked levels of the LOQ and 2.5 times the LOQ, the ratios of the 44 pesticides with recoveries of 60% to 120% were 88.64% and 100%, respectively. The LOQs of 200 pesticides with MRLs ≥0.05 mg/kg or without MRLs in fresh chilli did not exceed 0.025 mg/kg. The linear ranges of these 200 pesticides were 0.05-1.00. At spiked levels of the LOQ, twice the LOQ, and 10 times the LOQ, the ratios of the 200 pesticides with recoveries of 60% to 120% were 49.50%, 87.00%, and 89.50%, respectively. The linear correlation coefficients (*r* ^2^) of the 244 pesticides in fresh chilli were greater than 0.99. The LOQs of 222 pesticides in dried chilli were less than 0.15 mg/kg, and the linear ranges were 0.04-1.00 mg/L. The ratios of these 222 pesticides with *r* ^2^ greater than 0.99 was 95.46%. At spiked levels of the LOQ, twice the LOQ and 10 times the LOQ in dried chilli, the ratio of the 222 pesticides with recoveries of 60% to 120% were 72.52%, 73.42%, and 81.53%, respectively. The established screening and confirmation method was used to analyze 12 fresh chilli samples and 14 dried chilli samples. Eight pesticides were found in nine fresh chilli samples and three dried chilli samples, all of which were confirmed to be positive after manual identification. The concentrations of these pesticides were lower than the MRLs required by GB 2763-2019: National Food Safety Standard —— Maximum Residue Limits for Pesticides in Food. The results demonstrate that the established method is rapid, easy to execute, efficient, and reliable. It can be used for the high-throughput screening and quantitation of pesticide residues in fresh and dried chilli.

辣椒(*Capsicum* spp.)作为一种重要的蔬菜和调味品,在我国的种植面积超过200万公顷,在我国蔬菜作物中种植面积位居第一^[1]^。当前,应用于农业生产的农药种类超过1000种,我国于2020年发布实施的GB 2763-2019《食品安全国家标准食品中农药最大残留限量》中^[2]^,规定了辣椒中136种及干辣椒中70种农药残留的最大残留限量(MRL)。我国标准体系中检测这些具有MRL的农药残留方法主要包括气相色谱-质谱法(GC-MS)和液相色谱-串联质谱法(LC-MS/MS)等,例如GB/T 20769-2008和GB 23200.8-2016等现行标准虽然也能够对农药残留的种类有较大范围的覆盖,但其前处理一般耗时较长,且需标准品对照,用于定性测定的快速筛查时效率不高。四极杆/飞行时间质谱(Q-TOF/MS)作为典型的高分辨质谱技术,相比于四极杆、三重四极杆质谱,具有质量精度更高、通量更大、全质量数采集、数据库匹配检索等优势,定性确证能力更强,在多农残检测领域的应用越来越广^[3,4,5,6]^。QuEChERS前处理方法具有快速、方便、便宜、高效、耐用等优点,结合高分辨质谱技术能够对样品中农兽药残留^[7,8,9,10,11,12,13,14,15,16]^、生物毒素等^[17,18,19,20,21,22]^进行快速准确的筛查确证,欧盟、美国发布的EN 15662∶2018、AOAC Official Method 2007.01均采用QuEChERS前处理进行农药残留的分析,我国发布的GB 23200.113-2018也将QuEChERS方法作为农药残留的前处理方式。本文将QuEChERS前处理与GC-Q-TOF/MS结合,能够对辣椒及干辣椒中的农药多残留进行快速、准确的筛查。

## 1 实验部分

### 1.1 试剂与材料

农药混合标准品(批号S040267(含113种农药)、批号S039681(含109种农药)),质量浓度均为100 mg/L,溶剂为乙酸乙酯;环氧七氯B(批号S035355),质量浓度为100 mg/L,溶于甲醇,均购自日本岛津公司;其余44种固体农药标准品,纯度≥95%,购自美国A. Chemtek公司,均为认证标准物质。L-古洛糖酸内酯、D-山梨醇,分析纯,购自加拿大TRC公司,;乙腈、丙酮、乙酸等有机溶剂均为色谱纯(德国Merck公司),水为实验室所制得的一级水,提取盐包(Ⅰ:含4 g硫酸镁、1 g氯化钠、1 g柠檬酸钠、0.5 g柠檬酸氢二钠;Ⅱ:含6 g无水硫酸镁、1.5 g醋酸钠)、净化包(Ⅰ:含900 mg硫酸镁、150 mg *N*-丙基乙二胺(PSA); Ⅱ:含1200 mg硫酸镁、400 mg PSA、400 mg C_18_、200 mg石墨化炭黑(GCB))均购自上海安谱公司;鲜辣椒及干辣椒购自武汉各大实体超市及网上超市,产地包括湖北、四川、重庆、河南、陕西、山东等省份。

### 1.2 仪器及设备

7890B-7200B气相色谱-四极杆飞行时间质谱联用仪(GC-Q-TOF/MS,美国安捷伦公司),配有EI源;Talboys涡旋振荡仪(美国Troemner公司); Allegra X-15R离心机(美国Beckman Coulter公司); Milli-Q超纯水仪(德国Merck Millipore公司); N-evap 112氮吹仪(美国Organomation公司); ME204/02电子天平(瑞士梅特勒-托利多公司)。

### 1.3 标准溶液的配制

标准储备液:分别准确称取固态标准品10.0 mg,加入丙酮将其溶解并定容至10.0 mL,得到1.0 g/L的44种单标储备液;购买的液态混合标准溶液也作为部分参考标准物质的储备液,液态混合标准储备液分为A组和B组,分别有113种和109种。

混合标准中间液(10 mg/L):共3组,分别移取1 mL A组和B组标准储备液于10.0 mL棕色容量瓶中,加入丙酮定容至刻度,得到A组和B组各自的混合标准中间液;分别准确移取44种各农药单标储备液100 μL于10.0 mL棕色容量瓶中,加入丙酮定容至刻度,得到C组的混合标准中间液。

内标使用液(9 mg/L):准确移取0.9 mL环氧七氯B标准品(100 mg/L)于10.0 mL棕色容量瓶中,加入丙酮稀释定容至刻度。

### 1.4 样品前处理

鲜辣椒样品经切碎、匀浆,干辣椒样品经粉碎后充分混匀得到试样。采用GB 23200.113-2018中的前处理方式并做部分改进。称取10 g鲜辣椒试样(精确至0.01 g)于50 mL离心管中,加入10 mL -20 ℃乙腈和提取盐包Ⅰ,剧烈振荡1 min后4000 r/min离心5 min,吸取6 mL上清液于装有净化包Ⅰ的15 mL离心管中,涡旋混匀1 min后4000 r/min离心5 min,准确吸取2 mL上清液于15 mL离心管中,35 ℃下氮气吹至近干,加入20 μL内标使用液、50 μL分析保护剂(AP, 20 g/L L-古洛糖酸内酯和10 g/L D-山梨糖醇混合溶液)、1 mL丙酮复溶,过0.22 μm有机微孔滤膜后上机测定。称取2 g干辣椒试样(精确至0.01 g)于50 mL离心管中,加入10 mL水涡旋混匀1 min后静置30 min,加入15 mL乙腈-乙酸溶液(99∶1, v/v,使用前放入-20 ℃冰箱中冷冻过夜)和提取盐包Ⅱ,剧烈振荡1 min后4000 r/min离心5 min,吸取8 mL上清液于装有净化包Ⅱ的15 mL离心管中,涡旋混匀1 min后4000 r/min离心5 min,准确吸取2 mL上清液于15 mL离心管中,35 ℃下氮气吹至近干,加入20 μL内标使用液、1 mL丙酮复溶,过0.22 μm有机微孔滤膜后上机测定。

### 1.5 仪器条件

色谱 色谱柱为HP-5MS UI(30 m×0.25 mm×0.25 μm,美国安捷伦公司);初始柱温60 ℃,升温程序:60 ℃保持1 min, 40 ℃/min升温至120 ℃,再以5 ℃/min升温至310 ℃;载气:氦气,碰撞气:氮气,纯度均≥99.999%;载气流速0.997 mL/min;进样口温度:290 ℃;进样量:1 μL,不分流进样。

质谱 离子源:EI源,电压70 eV;离子源温度:230 ℃; GC-MS接口温度:280 ℃;数据扫描方式:Scan全扫描,分辨率≥20000,质量扫描范围*m/z* 45~550,采集速率:5质谱图/s, 200 ms/质谱图;数据采集存储格式:轮廓图和质心图;溶剂延迟:3.5 min。

### 1.6 定性和定量分析

数据采集和处理通过Agilent MassHunter Workstation(Quantitative Analysis 10.1和Qualitative Analysis 7.0)软件完成,通过MassHunter PCDL Manager(B.08.00)建立个人化合物数据库和谱图库(PCDL)。采用Quantitative Analysis 10.1运用建立好的PCDL库对GC-Q-TOF/MS采集的数据进行检索匹配定性分析。特征离子的检索匹配参数:保留时间最大偏差0.25 min,精确质量最大偏差20×10^-6^(20 ppm)。化合物检出判定条件:至少2个特征离子检出,综合得分>60分。检出的化合物采用基质匹配标准曲线进行定量分析。

## 2 结果与讨论

实验共选择244种农药作为研究对象,涵盖了GB 2763-2019中规定的辣椒及其制品中残留限量的79种(类)采用气相色谱或气相色谱-质谱联用法检测的农药,以及一些其他常见但未在辣椒及干辣椒中规定残留限量的农药,种类包括有机磷类、有机氯类、氨基甲酸酯类和拟除虫菊酯类等。在鲜辣椒中进行了244种农药的定性和定量方法的考察,包含44种在鲜辣椒中MRL≤0.05 mg/kg的农药及200种在鲜辣椒中无MRL规定或MRL>0.05 mg/kg的农药;在干辣椒中进行了222种农药的定性和定量方法的考察。

### 2.1 数据库的建立

采用GC-Q-TOF/MS对配制的混合标准溶液(0.5 mg/L)进行分析,在1.3.3节预设的实验条件下完成数据采集,得到特征离子精确质量数、保留时间等信息。每种化合物选择丰度最高的特征离子作为定量离子,另选择至少2个特征离子作为定性离子,保留时间、特征离子等参数见表1。244种农药中有10种(占比4.1%)农药采用分子离子作为定量离子,这10种农药的分子离子丰度在其特征离子丰度中为最高,其余的234种(占比95.9%)农药均采用碎片离子作为定量离子。244种农药的保留时间范围为5.563~37.047 min,保留时间分布情况见图1。收集每个化合物的名称、分子式、CAS号、保留时间、特征离子精确质量数和质谱图等信息,导入MassHunter PCDL Manager软件,建立PCDL库。

**表 1:** 244种农药化合物的分子式、精确质量数、特征离子、保留时间(t_R_)、线性相关系数(r^2^)和定量限(LOQ)

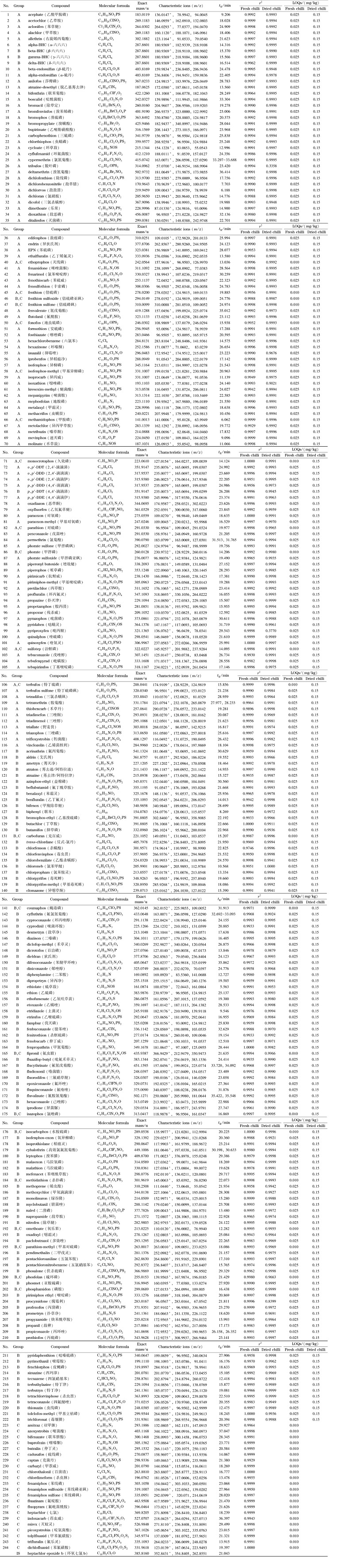

**图 1:**
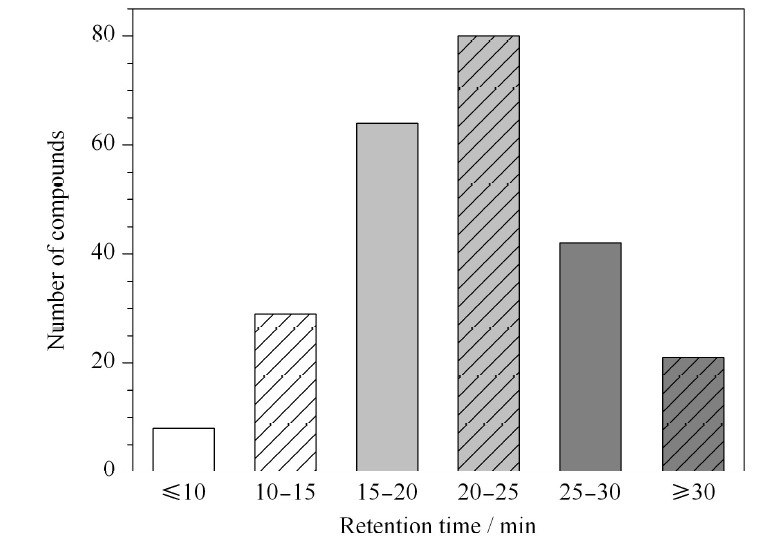
244种农药的保留时间分布

### 2.2 基质效应与实验条件的优化

2.2.1 基质效应

基质中的共提取物会干扰目标化合物的离子化,使目标化合物在仪器上的响应发生增强或抑制,这种干扰称为基质效应。分别用丙酮和空白基质提取液配制其中222种标准物质6个质量浓度(0.05、0.10、0.20、0.25、0.50、1.00 mg/L)的混合标准溶液,并建立标准曲线,比较两条曲线斜率的差异,从而判断基质效应的强弱,计算公式为:基质效应=[(基质匹配标准曲线斜率/溶剂标准曲线的斜率)-1]×100%。结果如图2所示,鲜辣椒基质的基质效应以基质增强效应为主,有200种农药表现出较强的基质增强效应,干辣椒基质的基质增强和基质抑制效应均存在,分别有59种和90种,可以看出两类基质中基质效应均显著存在。

**图 2:**
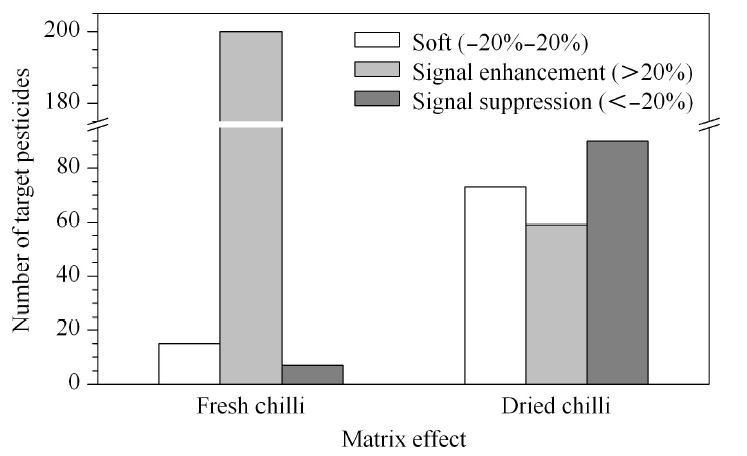
222种农药在辣椒中的基质效应分布

2.2.2 基质效应的补偿及实验条件的优化

目前常用的补偿基质效应的方法主要有基质净化、加入AP和基质匹配校准法等。基质匹配标准溶液应用最为广泛,但其要求有严格匹配的空白基质。AP已被证明能够有效解决有机磷和拟除虫菊酯等类农药残留分析中基质效应导致的诱导增强、峰形拖尾、灵敏度差、重现性差及线性差等问题,常用的AP有L-古洛糖酸内酯、D-山梨醇、乙二醇、聚乙二醇和橄榄油等^[22,23,24,25,26]^。分别用丙酮、加入AP的丙酮溶液和两种空白基质提取液配制质量浓度为100 μg/L的A、B两组的农药混合标准溶液,比较其中有机磷类农药响应和峰形的差异。结果显示,与未添加AP的标准溶液相比,添加AP的标准溶液中敌敌畏的响应有所降低,但添加AP的标准溶液中敌敌畏的峰形更好,且没有了拖尾现象;添加AP的标准溶液与鲜辣椒基质匹配标准溶液中敌敌畏的峰形、响应均相当;干辣椒基质匹配标准溶液中敌敌畏的响应远高于另外3种标准溶液中敌敌畏的响应,峰面积差别达10倍以上(见图3)。其他有机磷类农药如甲胺磷、乙酰甲胺磷等均具有类似的结果。说明AP能够用于鲜辣椒中补偿基质效应,这与文献报道^[22,23,24]^以及本实验室在进行果蔬鲜样的农药残留分析时的结果相一致,可以在没有对应的空白鲜辣椒基质时应用AP来进行农药的筛查分析;而在干辣椒中,AP补偿基质效应的效果有限,不适宜将AP用于干辣椒中农药的筛查分析,实际应用时,基质匹配标准溶液所得到的结果更为准确。基于此,本方法采用基质匹配校准法来补偿基质效应并对样品中的农药进行校准定量。

**图 3:**
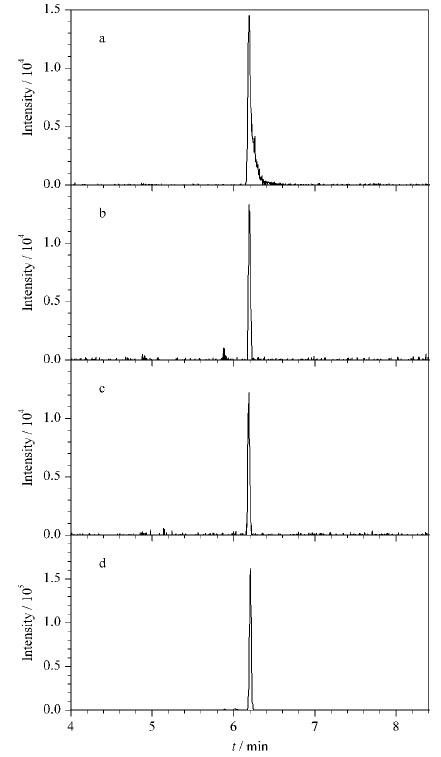
不同基质中敌敌畏(100 μg/L)的色谱图

在前处理过程中,对GB 23200.113-2018中的QuEChERS前处理方法进行了改进。保持整个提取过程的非高温环境,从而减少低挥发性农药的损失,采用过夜冷冻(-20 ℃)后的提取溶剂进行提取,防止提取过程中因盐包吸水放热使体系温度过高;离心机温度和氮吹水浴温度分别不超过4 ℃和35 ℃,氮气气流尽量放缓。在提取液及标准溶液中加入固定含量内标(0.18 μg环氧七氯B),比较在每次进样时内标物的响应变化,对设备稳定性进行校正,避免仪器波动对结果的干扰。

### 2.3 定性方法的建立

本文通过采集样品的轮廓质谱图(profile)获取分析物离子的峰形和原始分辨率信息,应用MassHunter软件对所采集的轮廓质谱图数据进行SureMass转换后,借助建立的PCDL库进行目标物的筛查匹配,主要筛查参数为保留时间最大偏差、目标物特征离子精确质量偏差范围、特征离子匹配数量。

保留时间偏差是筛查检测的一个重要参数。保留时间偏差设置过宽或过窄可能会造成假阳性或假阴性。在实际方法建立与筛查过程中发现,在设备、色谱柱、环境正常的情况下,色谱系统稳定性较高,保留时间偏差一般不超过0.1 min,本工作中这种稳定性也不受基质的影响,但因为同分异构体的存在,不同化合物对保留时间偏差设置的要求存在差异。氯氰菊酯、氯菊酯、氟氯氰菊酯配制于不同的混合标准溶液中,在实验条件下有完全相同的特征离子(见表1, *m/z* 163.0071, 206.0598, 127.0290),由图4可知,在31~34 min的窗口时间内,3种物质裂解得到的特征离子*m/z* 163.0071有共计10个未完全分离的色谱峰,如果保留时间偏差范围设置过宽,这些色谱峰相互之间容易有干扰导致假阳性和定量不准,增加后期人工手动鉴定和精确定量的工作量;同时,上述3种农药化合物以及丙环唑、氟胺氰菊酯、氯氟氰菊酯存在同分异构体,同分异构体之间因为特征离子相同、保留时间接近而导致色谱峰分离度不佳,保留时间偏差如果设置过窄将导致漏报使结果出现假阴性。针对上述情形,在对保留时间进行设定时,拟除虫菊酯类农药和丙环唑的保留时间设定为其所有异构体保留时间的中间点,偏差范围设置为±0.25 min。同时,在设备使用前后及时进行设备维护和保留时间锁定,保证目标物保留时间的稳定性。

**图 4:**
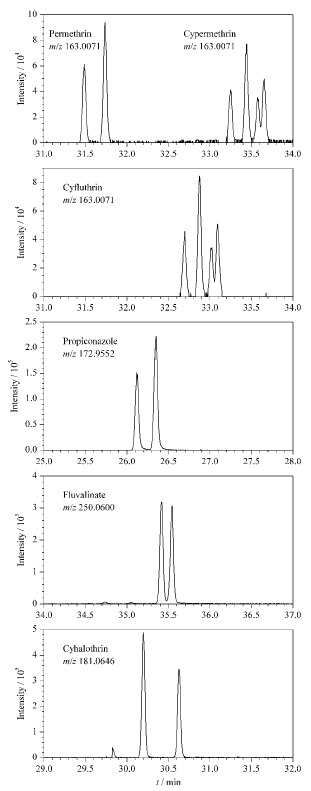
丙酮中丙环唑和5种拟除虫菊酯类农药标准溶液 (200 μg/L)的特征离子色谱图

目标物的精确质量作为筛查分析的关键参数,是高分辨质谱(HRMS)的最大优势,其偏差范围代表着筛查的选择性和特异性范围。农业农村部公告第312号和欧盟SANTE/12682/2019均要求目标物质量偏差低于5×10^-6^。本文实验发现,纯溶剂配制的目标农药质量偏差能够始终保持在5×10^-6^以内,但是两种空白基质提取液配制的目标农药质量偏差有较多大于5×10^-6^甚至10×10^-6^,例如甲胺磷在鲜辣椒和干辣椒中的质量偏差分别达到了11.4×10^-6^和12.1×10^-6^,如果将质量偏差范围设置为5×10^-6^以内,在借助建立的PCDL库进行筛查时,容易出现假阴性,基于此,本工作将目标农药的精确质量偏差阈值设置为20×10^-6^。

特征离子的数量是影响结果准确性的一项重要参数。表1中给出了利用参考标准物质所构建的数据库当中的化合物的3个特征离子信息,目标化合物裂解得到的片段数量一般多于3个,给出的3个特征离子为该化合物在实验条件下响应最高的特征离子,数据库中实际保留更多的特征离子数量以提高定性筛查的准确性。

为尽量降低假阴性,本文给出的保留时间和精确质量数阈值较宽,会在一定程度上增加假阳性报告的概率。在软件给出筛查结果后,还需手动对可疑检出农药进行人工鉴定和确证。包括考察目标物的峰形、离子相对丰度等。

### 2.4 定量方法的验证

本文在对样品进行复溶时加入了等量的内标,内标法所得校正曲线的线性相关系数和定量结果与外标法相比并无显著差异,但是通过每次进样时内标物响应的变化,能够对仪器的稳定性进行判断,必要时对设备进行维护保养。对样品进行测定时使用的校正曲线为内标法校正所得,用以抵消设备稳定性变化对定量结果带来的影响。鲜辣椒基质中244种农药的线性相关系数*r*^2^均大于0.99,线性范围在0.05~1.00 mg/L(200种)、0.01~1.00 mg/L(44种);干辣椒基质中222种农药的线性相关系数*r*^2^≥0.99的比例为95.46%,线性范围在0.04~1.00 mg/L,方法线性良好。

**表 2:** 244种农药在鲜辣椒中4个水平和干辣椒中3个水平下的加标回收率(*n*=5)

Sample	Number of pesticides added	Added/ (mg/kg)	Ratios/%			
			Recovery<60%	60%≤recovery≤120%		Recovery>120%
Fresh chilli	44^1)^	0.010	6.82	88.64		4.55
		0.025	0	100.00		0
	200^2)^	0.025	8.00	49.50		42.50
		0.050	5.50	87.00		7.50
		0.25	1.50	89.50		9.00
Dried chilli	222	0.15	15.32	72.52		12.16
		0.30	14.41	73.42		12.17
		1.5	6.76	81.53		11.71

1)MRL≤0.050 mg/kg; 2) MRL>0.050 mg/kg.

欧盟指南SANTE/12682/2019要求在一系列质量浓度水平进行添加回收试验以确定筛查限(SDL),农业农村部公告第312号中要求对于有参考标准品的化合物需进行方法中筛查检出限的补充,但未给出确定筛查检出限的方法。本文在进行定量方法验证时未参考欧盟指南单独考察SDL,主要确定了方法的定量限(LOQ)。考察了244种农药在鲜辣椒基质中的加标回收及检出情况,以信噪比(*S/N*)≥10对应的添加水平作为LOQ, MRL高于0.050 mg/kg或暂无限量值规定的农药有200种,其LOQ≤0.025 mg/kg; MRL不高于0.050 mg/kg的有44种,其LOQ≤0.010 mg/kg。干辣椒中农药残留限值均较高,0.040 mg/L基质标准溶液相当于样品含量0.15 mg/kg。考察222种农药在干辣椒基质中的加标回收及检出情况,以信噪比*S/N*≥10对应的添加水平作为LOQ,干辣椒基质中222种农药的LOQ≤0.15 mg/kg。具体化合物信息、分组信息、相关系数(*r*^2^)、LOQ等信息见表1。

对空白鲜辣椒和干辣椒样品进行农药的加标回收试验,每个水平重复5次(*n*=5)并计算回收率,结果见表2。鲜辣椒中MRL不高于0.050 mg/kg的44种农药在1倍和2.5倍LOQ即0.010和0.025 mg/kg两个水平进行加标回收试验,回收率在60%~120%的农药种类占比分别为88.64%和100%;鲜辣椒中MRL高于0.050 mg/kg或暂无限量值规定的200种农药在1倍、2倍和10倍LOQ即0.025、0.050和0.25 mg/kg 3个添加水平下,回收率在60%~120%的农药种类占比分别为49.50%、87.00%和89.50%。干辣椒中222种农药在1倍、2倍和10倍LOQ即0.15、0.30和1.5 mg/kg 3个添加水平下,回收率在60%~120%占比分别为72.52%、73.42%和81.53%。

在定量方法验证中,两种基质中农药的最低添加水平均不高于该农药在该基质中的MRL,在最低添加水平,虽然部分农药回收率不佳(回收率<60%或>120%),但所有添加的农药均能在1.6节的预设条件下完成定性筛查和确证。在实际应用中,若样品中筛查出的农药有MRLs规定,一方面可利用已建立的该农药的校正曲线进行定量分析,另一方面可再借助气相色谱-质谱联用仪、液相色谱-串联质谱仪等设备,对样品中残留的农药进行检测以进一步定量来满足日常监管工作的需要。

### 2.5 实际样品检测与分析

采用本文所建立的GC-Q-TOF/MS筛查确证技术对市售的辣椒样品进行244种农药残留的筛查分析。样品包含12个鲜辣椒样品和14个干辣椒样品,鲜辣椒样品购于武汉各实体超市,产地为山东和湖北两省;干辣椒样品为网购样品,产地涉及四川、河南、山东、重庆等8个省(市)。在9个鲜辣椒样品和3个干辣椒样品中筛查出8种农药化合物,经人工鉴定后确定了该筛查结果,表明这8种农药存在于相应样品中。同时,借助建立的标准曲线对这8种农药进行了定量,样品中农药残留筛查和定量结果见表3。

经软件筛查和人工鉴定,在鲜辣椒样品中,确证7种农药甲草胺、杀螨酯、烯唑醇、苯醚甲环唑、丙环唑、六氯苯、四氟醚唑共10项次;在干辣椒样品中,筛查确证2种农药速灭磷、苯醚甲环唑共4项次。上述农药用途包括杀菌剂(烯唑醇、苯醚甲环唑、丙环唑、六氯苯、四氟醚唑)、杀虫剂和杀螨剂(杀螨酯、速灭磷)、除草剂(甲草胺)。在5个鲜辣椒样品中分别检出甲草胺、烯唑醇、六氯苯、四氟醚唑和杀螨酯,含量均低于该农药LOQ;在2个鲜辣椒样品中检出丙环唑,含量均低于其LOQ;在3个干辣椒样品中检出速灭磷,含量均低于其LOQ;在3个鲜辣椒样品和1个干辣椒样品中检出苯醚甲环唑,含量分别为0.07、0.09、0.11和0.35 mg/kg,低于苯醚甲环唑在鲜辣椒和干辣椒中的MRL(鲜辣椒1 mg/kg,干辣椒5 mg/kg)。

筛查出苯醚甲环唑的4个样品中,苯醚甲环唑的定性及定量离子与建立的PCDL库中苯醚甲环唑的定性及定量离子的精确质量偏差均小于2×10^-6^,而在建立数据库时,空白溶剂标准溶液与基质匹配标准溶液中的苯醚甲环唑的特征离子精确质量差别超过了5×10^-6^,达到了8×10^-6^。说明基质效应虽然会对农药残留的质量稳定性造成影响,但在同一类基质中,这种影响可能具有相对稳定性,在建立筛查数据库时,若不能很好地排除背景干扰,可依据基质的差异建立基质匹配的数据库,或是适当加宽筛查条件中精确质量的偏差阈值,来保证数据库的准确有效应用。

**表 3:** 辣椒样品中农药残留的筛查及定量结果

Sample	Compound	MRL/(mg/kg)	Content/(mg/kg)	Frequency of detection
Fresh chilli	alachlor (甲草胺)	-	<0.025	1
	chlorfenson (杀螨酯)	-	<0.025	1
	diniconazole (烯唑醇)	-	<0.025	1
	difenoconazole (苯醚甲环唑)	1	0.07, 0.09, 0.11	3
	propiconazole (丙环唑)	-	<0.025	2
	hexachlorobenzene (六氯苯)	-	<0.025	1
	tetraconazole (四氟醚唑)	-	<0.025	1
Dried chilli	mevinphos (速灭磷)	-	<0.15	3
	difenoconazole (苯醚甲环唑)	5	0.35	1

-: No MRL in GB 2763-2019.

## 3 结论

本实验采用QuEChERS前处理方式并结合GC-Q-TOF/MS建立了鲜辣椒及干辣椒中的244种农药残留精确质量数据库及谱图库,能够对鲜辣椒和干辣椒进行筛查和定量分析,并对市售鲜辣椒和干辣椒样品进行了农药残留的筛查分析。方法快速、简便、高效、准确,在短时间内即可完成样品的前处理及上机操作,并结合所建立的涵盖精确质量数、保留时间、特征离子信息等信息的数据库,实现样品中244种农药残留的快速筛查和初步定量工作,并已在本实验室中获得初步应用,后续针对数据库的扩充和完善,也会让整个筛查方法更为全面,能够为食品安全监管提供有力的技术支撑。
